# The Impact of Dysmenorrhea on Quality of Life among Spanish Female University Students

**DOI:** 10.3390/ijerph16050713

**Published:** 2019-02-27

**Authors:** Elia Fernández-Martínez, María Dolores Onieva-Zafra, María Laura Parra-Fernández

**Affiliations:** Department of Nursing, Physiotherapy and Occupational Therapy, Faculty of Nursing of Ciudad Real, Universidad de Castilla-La-Mancha, 13071 Ciudad Real, Spain; elia.fernandez@uclm.es (E.F.-M.); marialaura.parra@uclm.es (M.L.P.-F.)

**Keywords:** dysmenorrhea, quality of life, university students

## Abstract

(1) Background: Primary dysmenorrhea, which is characterized by menstrual pain in the absence of a pelvic pathology, is one of the main reasons for gynecological consultation. This study aimed to assess the prevalence of dysmenorrhea in a sample of university students, as well as their quality of life, and to examine the most common methods used for alleviating symptoms. (2) Methods: The participants comprised 305 female university students with a mean age of 20.32 ± 3.19 years who completed a self-report survey comprising sociodemographic, gynecological and lifestyle questions. EuroQol-5 dimensions-5 levels (EQ-5D-5L) was used to measure quality of life. (3) Results: In total, 76% of the sample suffered from dysmenorrhea. Among the students who did not suffer from dysmenorrhea, a significantly greater proportion participated in activities such as jogging or Pilates on a regular basis (several times per week). Concerning quality of life, patients with dysmenorrhea showed significant differences on the pain/discomfort scale and on the total score for perceived quality of life. However, this perception showed no correlation with the VAS (visual analogue scale) pain scale. Additionally, 90.5% of students with dysmenorrhea used pharmacological treatment, and 80% self-medicated. (4) Conclusions: Dysmenorrhea represents a major problem among youth today and the impact on the quality of life (QoL) of patients is evident. Physical activity may alleviate symptoms of dysmenorrhea and this and other complementary treatments should be promoted within health services.

## 1. Introduction

Primary dysmenorrhea (PD) is defined as painful menstruation in the absence of a pelvic pathology and is one of the most common complaints in young women [1,2]. Secondary dysmenorrhea is defined as menstrual pain resulting from underlying pelvic pathologies [3]. Symptoms of PD include cramping pain in the lower abdominal area which may, or may not, radiate to the lower back and which is accompanied by a headache, nausea, tiredness, vomiting, irritability, diarrhea and an overall feeling of discomfort [4]. Dysmenorrhea may be classified as mild, moderate or severe, depending on the degree of pain experienced [5]. Aside from physical health, PD may also affect social aspects and a person’s everyday life [6]. The pain experienced by adolescents with PD can be very disabling and consequently affect the person’s mood [2]. Previous studies have demonstrated that young people affected by PD often miss work or school because of the discomfort, which, in turn, can have an important social and economic impact [4,7].

An excess of prostaglandins is liberated during menstruation. It is thought that this may be the cause of incapacitating pain, as an excess of prostaglandins may lead to excessive uterine contractions which, in turn, may result in hypoxia and ischemia of the uterus, and lead to the typical pain of PD [8]. Non-steroidal anti-inflammatory drugs (NSAIDs) are the pharmacological therapy of choice for patients with PD [9]. However, most patients with PD do not seek medical help, or that of other health professionals. Instead, they self-medicate or seek alternative solutions. The continued use of such self-medication without a medical prescription or, at the very least, a professional assessment, can also result in secondary effects [10]. In addition, it is important to note that, over recent years, there has been a major demand for new, complementary or integrative therapies which coexist alongside traditional medicine [11]. In this sense, several studies have proven that different complementary therapies, such as yoga, acupressure or herbal medicine, among others, may contribute towards an improvement in the symptoms of PD and therefore lead to improvements in overall quality of life (QoL) [11,12,13,14]. The findings reported by Mc Govern et al. suggest that yoga is a safe and effective treatment for improving QoL through reduction of pain, stress, anxiety, depression and insomnia, a conclusion also made by other authors [15,16]. In a systematic review on complementary medicine for the treatment of dysmenorrhea, Sharghi et al. [17] concluded that medicinal plants, drugs, acupuncture and acupressure appear to suppress pain in these patients. According to a systematic review by Song et al. [18], aromatherapy was found to be an effective intervention for reducing menstrual pain in dysmenorrhea. Likewise, the use of supplements has also spread as a complementary treatment for menstrual pain associated with dysmenorrhea [19]. 

Considering the high prevalence of PD and its effect on the QoL of women, the promotion of a healthy lifestyle and self-care in young women should be one of the objectives of socio-sanitary care. The nursing profession should therefore embrace comprehensive interventions or support groups by including complementary therapies that may benefit the quality of life of these patients.

Until now, few studies have examined the prevalence of dysmenorrhea among samples of university students in Spain [20,21]; however, none of these have focused on the perception of quality of life of these patients and which complementary methods they use or fail to use for the relief of their symptoms. Therefore, the aim of this study was to explore the prevalence of dysmenorrhea in a sample of university students, as well as to evaluate students’ quality of life, and examine the complementary methods applied or known to them for alleviating their symptoms.

## 2. Materials and Methods 

### 2.1. Study Design, Setting and Participants

This was an observational cross-sectional and descriptive study performed on female nursing students of the Faculty of Nursing of Ciudad Real (Spain). The exclusion criterion was a failure to agree to participate in the study. All students over the age of 18 that enrolled in the 2017/2018 academic year of this faculty were invited to participate in the study. No prior sample size calculation was performed. There were two eligibility criteria for this study: Being a female student enrolled in a degree course taught at the university, and be present when the researcher visited the classroom in person for data collection. Participation was voluntary and participants received no financial incentive. Students who were participating in academic exchanges were excluded, along with those who declined to participate.

The data were gathered using an ad hoc survey designed by the research group based on the available literature on the topic of study. The survey comprised sociodemographic questions, questions on lifestyle, specific questions on dysmenorrhea and methods for pain relief and was complemented with the EuroQol-5D quality of life scale in the Spanish validated version. The survey was distributed online using the Google Forms platform. 

The Spanish version of the EQ-5D scale was used to evaluate QoL. The EQ-5D instrument has two components. The EQ-5D Index quantifies health state in terms of five dimensions (mobility, self-care, daily activity, pain or discomfort, and anxiety or depression). Each item is rated on a three-point scale (never = 0, sometimes = 1, and always = 2). EQ-5D also included a global health state measurement on perceived quality of life based on a visual analogue scale (VAS) ranging from 0 to 100, in which 0 indicated the worst possible health status and 100 indicated the best possible health status [22,23]. 

The study protocol was approved by the Clinical Research Ethics Committee at the Ciudad Real General Hospital (approval number C-105). All procedures were made in accordance with the Helsinki Declaration. Permission to conduct the study was obtained from the management of the Nursing Faculty. Before data were collected, all students were informed of the purpose of the study and informed consent was obtained. In addition, all participants were assured that their anonymity and confidentiality would be maintained and that they were entitled to drop out of the study at any time.

### 2.2. Data Analysis

Statistical analyses were performed using SPSS for Windows version 23.0 (Statistical Package for Social Sciences Inc., Chicago, USA) after the data were exported to an excel document of the Microsoft Office package. A descriptive analysis was performed, whereby quantitative variables were expressed using the mean and standard deviation, and qualitative variables were examined using frequencies and percentages. The normality of the distribution of the quantitative variables was analyzed using the Kolmogorov Smirnov test, and thereafter, the most suitable bivariate was tested according to each case. The Chi-squared test, Fisher’s exact test and the U Mann Whitney test were used for comparing the sociodemographic variables in women with and without dysmenorrhea. Furthermore, the Chi-squared test and Fisher’s exact test were used to compare the different dimensions of EuroQol in the same groups of participants, i.e., women with and without dysmenorrhea. To compare scores of perceived quality of life among women with and without dysmenorrhea, the U Mann Whitney test was used. In addition, the Kruskal—Wallis test was used in students categorized by BMI according to World Health Organization (WHO) and in students categorized by menstrual pain according to VAS. To study the correlation between perceived quality of life, BMI and hours of sleep, the Spearman’s Rho test was used. A *p* value of <0.05 was considered to be statistically significant.

## 3. Results

### 3.1. Sociodemographic Characteristics and Lifestyle of Respondents

A total of 302 female students completed the survey. In our study the mean age of the students was 20.32 ± 3.19 years and the body mass index was 21.65 ± 3.22. Of the sample, 23.5% (n = 71) did not suffer from dysmenorrhea, compared to 76.5% (*n* = 231) who reported having dysmenorrhea. Regarding their lifestyle, in relation to sports activities performed, 33.4% (*n* = 101) of the sample acknowledged that they had no weekly routine with regard to exercising, whereas 31.8% (*n* = 96) stated that they generally perform less than three hours of exercise a week and 34.8% (*n* = 105) indicated that they usually exercise three or more hours per week. The mean duration of physical exercise per week was 2.66 ± 2.60 hours. The exercise that women practiced the most was running, as stated by 36.1% (*n* = 109), followed by cycling, practiced by 28.1% (*n* = 85). The least practiced sports by the women who participated in the survey were swimming, practiced by 7.6% (*n* = 23), Pilates, practiced by 4.6% (*n* = 14) and yoga, practiced by 2% (*n* = 6). Regarding amount of rest, the mean number of daily hours of sleep was 7.09 ± 0.96. Up to 69.9% (*n* = 211) commonly slept less than eight hours per day, compared to 30.1% (*n* = 91) who slept eight hours or more per day. Among the women who did not suffer from dysmenorrhea, there were significantly more women who regularly ran (*p* = 0.037) or did Pilates (*p* = 0.025). However, no statistically significant differences were found among the groups of women regarding the total hours of exercise or hours of sleep (Table 1).

### 3.2. Quality of Life of the Participants

Regarding the dimensions of EuroQol, 3% (n = 9) of participants had mobility problems, 0.7% (*n* = 2) had personal care problems, 5.3% (*n* = 16) manifested problems regarding daily activities, 16.5% (*n* = 50) had discomfort/pain and 24.2% (*n* = 73) had problems related to anxiety/depression. When analyzing the proportion of women who presented (or not) problems in the different EuroQol dimensions and comparing these with the dysmenorrhea and non-dysmenorrhea sufferers, significant differences were identified regarding problems in the dimension of pain and discomfort in women with dysmenorrhea (*p* = 0.036). However, for the remaining dimensions, statistically significant differences were not found (Table 2).

**Figure 1:**
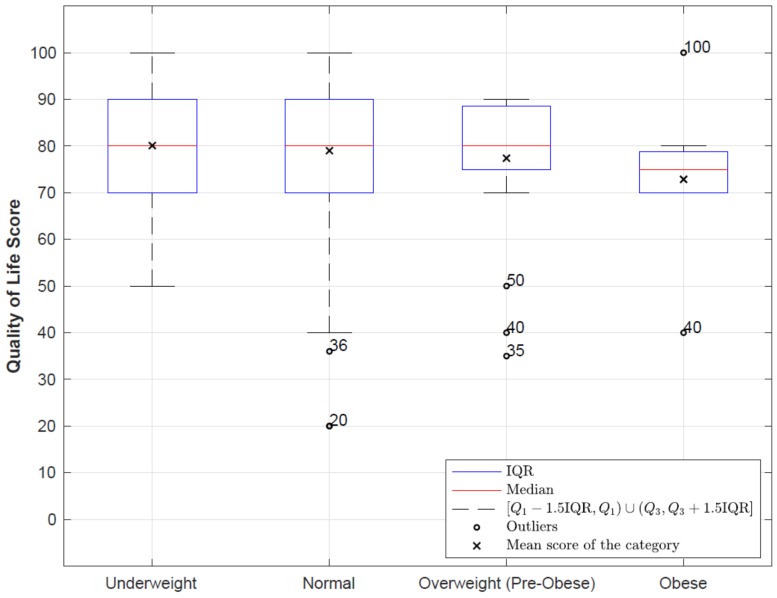
Comparison of mean scores on the perception of quality of life using EuroQol5-D among women with and without dysmenorrhea.

**Figure 2:**
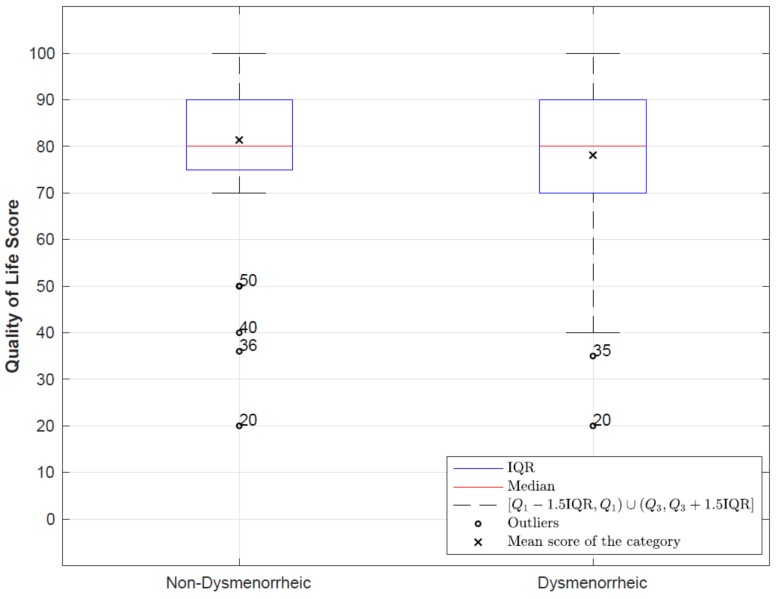
Comparison of the perceived quality of life in the different groups according to the WHO BMI classification.

Regarding the self-perception scores of EuroQol, the mean score of the study sample was 78.87 ± 14.74 with a minimum of 20 and a maximum of 100. Statistically significant differences were found (*p* = 0.028) when comparing the mean perceived QoL using EuroQol-5D from 0—100 among women with, and without, dysmenorrhea. Women with dysmenorrhea had a lower mean score (78.11 ± 1.33) when compared to the score of those who did not suffer from dysmenorrhea (81.37 ± 15.83) (Figure 1). There was no correlation between the hours of sleep of each student and their perceived QoL (r = 0.023, *p* = 0.690). However, a statistically significant inverse correlation was confirmed between the BMI score and perceived QoL (*r* = −0.118, *p* = 0.040). The BMI values (calculated using self-reported height and weight) were interpreted based on the BMI classification published by the World Health Organization, whereby a BMI of below 18.5 was categorized as underweight, of 18.5–24.99 as normal, of 25.0–29.99 as overweight (pre-obese), and of 30 and above as obese [24]. Nonetheless, when comparing the mean perceived QoL according to the BMI groups of WHO, these differences were not statistically significant: Low weight (80.09 ± 14.81), normal weight (79.07 ± 14.78), overweight (77.39 ± 14.28) and obesity (72.86 ± 14.77) (Figure 2).

### 3.3. Pain and Quality of Life in Participants with Dysmenorrhea 

In women who suffered from dysmenorrhea, the mean intensity of pain in the three previous cycles, prior to the consultation, was 6.90 ± 1.70, with the mean score being three and the maximum score being 10. When categorizing VAS, in accordance with previous bibliography [25], as mild (1–3), moderate (4–6) and severe (7–10), 66.2% (*n* = 153) had severe dysmenorrhea, 30.7% (*n* = 71) had moderate dysmenorrhea and only 3% (*n* = 7) had mild dysmenorrhea. 

The perceived QoL of women with dysmenorrhea had no correlation with VAS scores when grouping subjects according to the different levels of intensity of pain. No significant differences were found in the total score (see Table 3) or in the EuroQol dimensions (see Table 4).

### 3.4. Methods of Pain Relief Employed

Up to 90.5% (*n* = 209) of the women with dysmenorrhea were taking analgesics to alleviate the pain, representing the most commonly used pharmacological approach. Of the 209 women who used analgesia, 84.21% (*n* = 176) did so without any type of health prescription and only 15.79% (*n* = 33) had previously received a medical assessment and prescription for the analgesia. The non-pharmacological methods that the students with dysmenorrhea most frequently used were: Antalgic postures, 92.2% (*n* = 213); heat applications, 61% (*n* = 141); evening primrose oil, 57.1% (*n* = 132); television and music, 46.8% (*n* = 108); local massage, 41.6% (*n* = 96); relaxation techniques, 24.2% (*n* = 56); meditation, 5.2% (*n* = 12); music therapy, 4.3% (*n* = 10); acupressure, 3.5% (*n* = 9); vitamin supplements, 3% (*n* = 7); Transcutaneous Electricla Nerve Stimulation (TENS), 1.7% (*n* = 4); aromatherapy, 1.3% (*n* = 3) and acupuncture, 0.9% (*n* = 2) (see Table 5).

## 4. Discussion

We found a high prevalence of dysmenorrhea among our sample of university students (76.5%), which is in line with other international studies among young female university students [26,27,28]. The worldwide prevalence of dysmenorrhea, however, ranges between 50% and 90% [2,29,30]; this disparity is possibly because the diagnosis of dysmenorrhea varies according to the age of women included in study samples. Furthermore, prevalence reports may be influenced by self-report bias. 

The present study revealed that there were more women who regularly practiced activities such as running and Pilates among those who did not suffer from dysmenorrhea. These findings are in line with a study by Muluneh et al. [31] in which physical activity was found to be a protective factor for the occurrence of dysmenorrhea, whereas in a review by Geenen at al. [32], different physical activities, including Pilates, were considered to be potentially beneficial for the management of pain and the improvement of QoL among adults who experienced chronic pain. A number of studies support the evidence that physical activity can contribute towards the improvement of the symptoms associated with dysmenorrhea [1,33], and, in general, many doctors recommend that these patients perform physical activity. However, findings are still inconclusive regarding which is the best sport to practice, or how much time should be devoted to it in order to notice the benefits on the symptoms of dysmenorrhea, due to the considerable heterogeneity of the same sample [1,32]. A previous study by Dehanvi et al. [25] based on an intervention of eight weeks of aerobic exercise demonstrated a significant improvement in the case of women with dysmenorrhea compared to the control group who had similar demographic characteristics and who did not practice any type of sport. In a study by Ortiz et al. [34], with a mixed intervention of relaxation exercises, Kegel exercises, stretches and jogging, and with a total duration of 50 minutes, three times per week, an improvement on the level of pain was demonstrated by patients with dysmenorrhea when compared to the control group who did not practice any type of sport. Considering that sports practice appears to be a protective factor for the onset of dysmenorrhea, clinicians should inform women that physical activity may be an effective treatment for primary dysmenorrhea, although high-quality trials are needed before this can be proven. [1]

Studies assessing the perception of QoL in women with dysmenorrhea clearly show a decrease of the pain in women affected by this pathology. In our study, it is important to note that significant differences were only found among women with and without dysmenorrhea in the dimension pain/discomfort, coinciding with other studies where pain was noted by most women as being the most incapacitating factor and, therefore, the only one responsible for the decreased QoL [35]. Considering that our study population consisted of young women who attended university, the impact on their QoL clearly equals a reduction in their ability to participate socially, thus increasing absenteeism during school activities and, consequently, having a negative impact on academic achievements [36]. In various epidemiological studies, a relationship has been found between BMI and suffering from dysmenorrhea [29,37]. In our study, by grouping the women according to their BMI and correlating this with quality of life, the results were non-significant; however the scores were worse among those patients with a lower BMI.

Non-steroidal anti-inflammatory drugs are used as the first-line treatment of primary dysmenorrhea [38]. In our study, 90% of women with dysmenorrhea self-medicated, with 80% of these doing so without any type of medical prescription. A number of studies suggest that self-medication of this patient population is high [39]. In contrast, in a study by Wong et al. on a population of adolescent girls in Hong Kong, only 18% of those surveyed affirmed that they self-medicated. This low result was attributed to the fact that, in China, even self-medication requires at least a prescription or medical advice [40]. In Spain, the only requisite to access this type of medication is to be over the age of 18, without the need for any type of advice or prior prescription. This would explain the high number of girls who self-medicate. However, it is important to avoid justifying self-medication just because of accessibility; instead, we should seek the specific causes underlying why self-medication exists. Some authors suggest that these patients usually seek help when the impact of the illness on their quality of daily life becomes unmanageable and not when the first symptoms appear [41]. Therefore, in some cases, a positive therapeutic approach could prevent excessive self-medication. Furthermore, regarding other treatment approaches, these may vary across cultures. For example, Eastern cultures are more likely to use methods such as acupuncture and traditional remedies. In a study performed in Japan [42] on the treatment preferences of girls with dysmenorrhea, the data revealed that, despite the fact that self-medication was still at the top of preferred treatments (chosen by 58% of participants), 12% of the sample stated that they preferred being treated using traditional Chinese medicine, compared to less than 1% of the participants in our survey, who stated that they would use acupuncture. 

The goal for the treatment of dysmenorrhea is to apply evidence-based treatments that include integrative therapies in order to achieve the best possible clinical outcome. However, not all the literature to date is conclusive in this sense, either because there is still a need for further studies to scientifically support the results or because, when comparing the few studies available, these present limitations such as randomization bias due to the limited number of studies, or the heterogeneous nature of the sample. Therefore, it remains uncertain whether the existing evidence is sufficiently rigorous. The use of anti-inflammatory drugs is a worldwide trend; however other treatments may vary. For example, acupuncture or acupressure treatments depend very much on the practicing therapist, and the application methods of aromatherapy and the types of oils used are variable [43]. The authors of the Cochrane systematic review on the use of acupuncture for dysmenorrhea concluded that there is insufficient evidence to demonstrate whether or not acupuncture or acupressure is effective for the treatment of primary dysmenorrhea [44]. Nevertheless, this should not be considered a hindrance, but rather, this may be an incentive to continue researching effective results for this complaint, not only regarding these treatments but also the combination of them with those that have already been scientifically proven. In this sense, researchers should seek consensus when designing studies by employing sound methodologies to improve the quality and evidence of the results. It is important to reinstate that one of the advantages of these treatments is the lack of side effects [17].

The findings from this study should be interpreted in light of several limitations. This study used retrospective self-reporting to obtain information regarding menstrual symptoms. This type of reporting may be prone to report bias. Furthermore, our study population included women from a single faculty; therefore, future studies should be performed in other study populations. Moreover, the generalization of findings to other countries may be limited.

## 5. Conclusions

Dysmenorrhea represents a major problem among youth today and the impact on the QoL of patients is evident. Therefore, further research is necessary in order to seek new integrative therapeutic methodologies to offer a wider choice of treatments, yet without overlooking the short and long term implications to patients’ symptoms. The nursing profession should become involved in the promotion of healthy lifestyles in the younger population (e.g., regarding sports and nutrition), as well as introduce the use of complementary therapies.

## Figures and Tables

**Table 1:** Comparison of variables among women with and without dysmenorrhea.

Lifestyle Variables		Dysmenorrhea	No Dysmenorrhea	*p* value
**Age** **(Mean ± SD)**		20.30 ± 3.36	20.41 ± 2.57	0.373 ^1^
BMI (Mean ± SD)		21.55 ± 3.31	21.99 ± 2.94	0.146 ^1^
Hours of sleep/day (Mean ± SD)		7.09 ± 0.95	7.11 ± 1.01	0.945 ^1^
Hours of exercise/week (Mean±SD)		2.57 ± 2.68	2.97 ± 2.30	0.069 ^1^
Running (*n* (%))	Yes	76 (32.9%)	33 (46.5%)	**0.037** ^2^*
	No	155 (67.1%)	38 (53.5%)	
Cycling (*n* (%))	Yes	63 (27.3%)	22 (31.0%)	0.543 ^2^
	No	168 (72.7%)	49 (69.0%)	
Swimming (*n* (%))	Yes	16 (6.9%)	7 (9.9%)	0.415 ^2^
	No	215 (93.1%)	64 (90.1%)	
Pilates (*n* (%))	Yes	7 (3%)	7 (9.9%)	**0.025** ^3^*
	No	224 (97%)	64 (90.1%)	
Yoga (*n* (%))	Yes	4 (1.7%)	2 (2.8%)	0.628 ^3^
	No	227 (98.3%)	69 (97.2%)	

^1^ U Mann Whitney test. ^2^ Chi-squared test.^3^ Fisher’s exact test. * *p* > 0.05.

**Table 2:** Percentage of the sample of women with, or without, dysmenorrhea who presented problems in one of the EuroQol dimensions.

EuroQol dimensions		Dysmenorrhea *n* (%)	No dysmenorrhea *n* (%)	*p* value
**Mobility**	No problem	225 (97.4%)	68 (95.8%)	0.442 ^1^
	Problem	6 (2.6%)	3 (4.2%)	
Personal care	No problem	230 (99.6%)	70 (98.6%)	0.416 ^1^
	Problem	1 (0.4%)	1 (1.4%)	
Daily activities	No problem	218 (94.4%)	68 (95.8%)	0.771 ^1^
	Problem	13 (5.6%)	3 (4.2%)	
Pain/discomfort	No problem	187 (81%)	65 (91%)	0.036 ^2^*
	Problem	44 (19%)	6 (8%)	
Anxiety/depression	No problem	172 (74.5%)	57 (80.3%)	0.316 ^2^
	Problem	59 (25.5%)	14 (19.7%)	

^1^ Fisher’s exact test. ^2^ Pearson’s chi-squared test.* *p* < 0.05.

**Table 3:** Comparison of the mean total scores of quality of life in women, grouped according to the intensity of dysmenorrhea.

Intensity	Quality of Life EuroQol5-D (Mean ± SD)	*p*-value ^1^
Mild (1–3)	85.29 ± 9.07	0.181
Moderate (4–7)	76.72 ± 14.23	
Severe (8–10)	78.42 ± 14.52	

^1^ *p* < 0.05.

**Table 4:** Dimensions of Euroqol identified as problems in women grouped according to the intensity of their dysmenorrhea.

Euroqol Dimensions/VAS Categories		Mild *n* (%)	Moderate *n* (%)	Severe *n* (%)	Total *n* (%)	*p* value
**Mobility**	No problem	7 (100%)	69 (97.2%)	149 (97.4%)	225 (97.4%)	0.905
	Problem	0 (0%)	2 (2.8%)	4 (2.6%)	6 (2.6%)	
Personal care	No problem	7 (100%)	71 (100%)	152 (99.3%)	230 (99.6%)	0.775
	Problem	0 (0%)	0 (0%)	1 (0.7%)	1 (0.4%)	
Daily activities	No problem	6 (85.7%)	70 (98.6%)	142 (92.8%)	218 (94.4%)	0.131
	Problem	1 (14.3%)	1 (1.4%)	11 (7.2%)	13 (5.6%)	
Pain/discomfort	No problem	4 (57.1%)	58 (81.7%)	125 (81.7%)	187 (81%)	0.265
	Problem	3 (42.9%)	13 (18.3%)	28 (18.3%)	44 (19%)	
Anxiety/depression	No problem	6 (85.7%)	58 (81.7%)	108 (70.6%)	172 (74.5%)	0.163
	Problem	1 (14.3%)	13 (18.3%)	45 (29.4%)	59 (25.5%)	

**Table 5:** Use of pain relief methods in women categorized by their pain intensity in relation to VAS.

Pain Relief Methods	Yes/No	CATEGORIZED VAS		
		Mild *n* (%)	Moderate *n* (%)	Severe *n* (%)
Analgesics	No	2 (28.6%)	13 (18.3%)	7 (4.6%)
	Yes, self-medicates	5 (71.4%)	54 (76%)	117 (16.5%%)
	Yes, with a prescription	0 (0%)	4 (5.6%)	29 (119%)
Music therapy	No	6 (85.7%)	69 (97.2%)	146 (95.4%)
	Yes	1 (14.3%)	2 (2.8%)	7 (4.6%)
Massage	No	5 (71.4%)	43 (60.6%)	87 (56.9%)
	Yes	2 (28.6%)	28 (39.4%)	66 (43.1%)
Acupuncture	No	7 (100%)	70 (98.6%)	152 (99.3%)
	Yes	0 (0%)	1 (1.4%)	1 (0.7%)
Acupressure	No	7 (100%)	70 (98.6%)	146 (95.4%)
	Yes	0 (0%)	1 (1.4%)	7 (4.6%)
Walking	No	5 (71.4%)	59 (83.1%)	110 (71.9%)
	Yes	2 (28.6%)	12 (16.9%)	43 (28.1%)
TV or music	No	3 (42.9%)	44 (62%)	76 (49.7%)
	Yes	4 (57.1%)	27 (38%)	77 (50.3%)
Antalgic postures	No	1 (14.3%)	7 (9.9%)	10 (6.5%)
	Yes	6 (85.7%)	64 (90.1%)	143 (93.5%)
Heat	No	4 (57.1%)	38 (53.5%)	48 (31.4%)
	Yes	3 (42.9%)	33 (46.5%)	105 (68.6%)
Evening primrose oil	No	3 (42.9%)	28 (39.4%)	68 (44.4%)
	Yes	4 (57.1%)	43 (60.6%)	85 (55.6%)
Meditation	No	7 (100%)	66 (93%)	146 (95.4%)
	Yes	0 (0%)	5 (7%)	7 (4.6%)
TENS	No	7 (100%)	69 (97.2%)	151 (98.7%)
	Yes	0 (0%)	2 (2.8%)	2 (1.3%)
Aromatherapy	No	7 (100%)	71 (100%)	150 (9.8%)
	Yes	0 (0%)	0 (0%)	3 (2%)
Vitamin supplements	No	7 (100%)	71 (100%)	146 (95.4%)
	Yes	0 (0%)	0 (0%)	7 (4.6%)
